# The Threonine Protease Activity of Testes-Specific Protease 50 (TSP50) Is Essential for Its Function in Cell Proliferation

**DOI:** 10.1371/journal.pone.0035030

**Published:** 2012-05-04

**Authors:** Yu-Yin Li, Yong-Li Bao, Zhen-Bo Song, Lu-Guo Sun, Ping Wu, Yu Zhang, Cong Fan, Yan-Xin Huang, Yin Wu, Chun-Lei Yu, Ying Sun, Li-Hua Zheng, Guan-Nan Wang, Yu-Xin Li

**Affiliations:** 1 National Engineering Laboratory for Druggable Gene and Protein Screening, Northeast Normal University, Changchun, China; 2 Research Center of Agriculture and Medicine Gene Engineering of Ministry of Education, Northeast Normal University, Changchun, China; 3 Institute of Genetics and Cytology, Northeast Normal University, Changchun, China; Cincinnati Children's Hospital Medical Center, United States of America

## Abstract

**Background:**

Testes-specific protease 50 (TSP50), a newly discovered threonine enzyme, has similar amino acid sequences and enzymatic structures to those of many serine proteases. It may be an oncogene. TSP50 is up-regulated in breast cancer epithelial cells, and ectopic expression of TSP50 in TSP50-deficient Chinese hamster ovary (CHO) cells has been found to promote cell proliferation. However, the mechanisms by which TSP50 exerts its growth-promoting effects are not yet fully understood.

**Methodology/Principal Findings:**

To delineate whether the threonine protease activity of TSP50 is essential to its function in cell proliferation, we constructed and characterized a mutant TSP50, called TSP50 T310A, which was identified as a protease-dead mutant of TSP50. By a series of proliferation analyses, colony formation assays and apoptosis analyses, we showed that T310A mutation significantly depresses TSP50-induced cell proliferation in vitro. Next, the CHO stable cell line expressing either wild-type or T310A mutant TSP50 was injected subcutaneously into nude mice. We found that the T310A mutation could abolish the tumorigenicity of TSP50 in vivo. A mechanism investigation revealed that the T310A mutation prevented interaction between TSP50 and the NF-κBIκBα complex, which is necessary for TSP50 to perform its function in cell proliferation.

**Conclusion:**

Our data highlight the importance of threonine 310, the most critical protease catalytic site in TSP50, to TSP50-induced cell proliferation and tumor formation.

## Introduction

Testes-specific protease 50 (TSP50) was discovered on a hypomethylated DNA fragment isolated from human breast cancer cells using the methylation-sensitive representational difference analysis technique [1]. TSP50 transcripts have been detected predominantly in human testes and are not visible in other normal tissues. However, most patients with breast cancer or colorectal carcinoma show abnormal TSP50 activation and expression [2], [3], [4]. Downregulation of TSP50 expression has been found to reduce cell proliferation and colony formation [5]. Our previous studies have revealed that the overexpression of TSP50 in CHO cells can markedly promote cell proliferation and colony formation in vitro and stimulate tumor formation in nude mice [6]. These results indicate that TSP50 could be an oncogene.

TSP50 is a member of the peptidase S1 family of serine proteases. Serine proteases carry out a diverse array of physiological and cellular functions, ranging from digestive and degradative processes to blood clotting, cellular and humoral immunity, embryonic development, fibrinolysis, fertilization, protein processing, and tissue remodeling [7]. Serine proteases have been classified into evolutionarily unrelated clans, which have been subdivided into families of proteases whose homology can be established statistically [8], [9]. Serine proteases are characterized by an active serine in their catalytic site. Two other residues, a histidine and an aspartate, are associated with the active serine, constituting what is referred to as the “catalytic triad” in many families of serine proteases, including the trypsin (S1), subtilism (S8), prolyl oligopeptidase (S9), and serine carboxypeptidase (S10) families [9], [10]. The positions of these residues are more or less conserved, with the codons for the catalytically essential histidine and serine being almost immediately adjacent to their exon boundaries [8], [9].

TSP50 is homologous to many serine proteases and contains a peptidase S1 domain (93–358). The amino acid sequence alignment of TSP50 with seven serine proteases showed that it shares 26–36% identity with those proteases. Enzymatic structures are also very similar [2], [11]. However, the catalytic triad of TSP50 is different from that of traditional serine proteases. TSP50 contains the first two sites of the catalytic triad, His and Asp, at positions 153 and 206, respectively. However, the third site, Ser, at position 310, is replaced by threonine. In this way, TSP50 represents a novel classification because of its Thr310 residue substitution, which may play an important catalytic role [11].

The threonine catalytic site of TSP50 is crucial to its protease activity [11]. However, whether this threonine catalytic site is necessary to the ability of TSP50 to promote cell proliferation remains to be determined. In this study, we used site-directed mutagenesis and a series of the cell proliferation and tumorigenicity assays to show that the TSP50 T310A mutation could abolish the cell-proliferation-promoting function of TSP50. Further studies demonstrated that the TSP50 T310A mutation could destroy the interaction between TSP50 and the NF-κB:IκBα complex, which is necessary for TSP50 to perform its function in cell proliferation. These results indicate that threonine 310, the most critical protease catalytic site of TSP50, is essential to the interaction between TSP50 and the NF-κB:IκBα complex and therefore TSP50’s role in cell proliferation. The dominant negative mutant allele constructed in this study may further understanding of the TSP50 and may be useful in cancer therapy.

## Results

### Structure of TSP50

**Figure 1:**
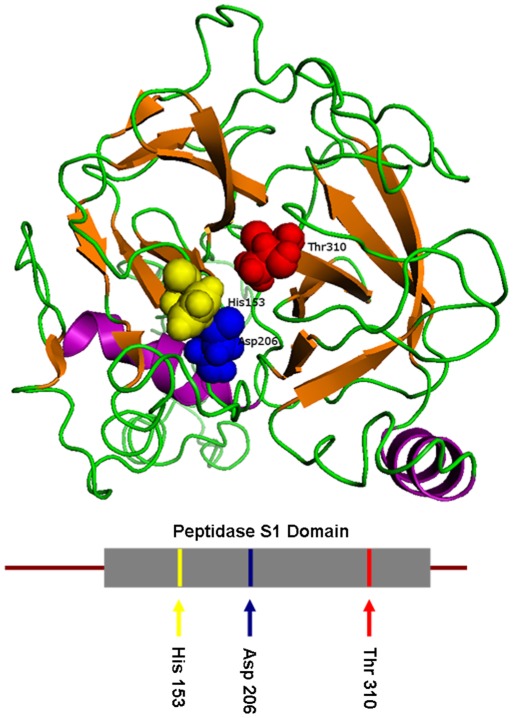
Predicted structure of human TSP50. α-helices (pink), β-sheets (orange), and loops (green) are labeled in this predicted structure of homo-sapiens TSP50 (113–369). The positions of amino acids within the catalytic triad are shown. The catalytic triad consists of a histidine at position 153, an aspartic acid at position 206, and a threonine, the most critical protease catalytic site of TSP50, at position 310. This diagram was prepared using PyMOL.

The structure of human TSP50 was predicted and visualized in PyMOL. As shown in Figure 1, TSP50 consists of a peptidase chymotrypsin (S1) domain (93–358), which contains the catalytic triad. The catalytic triad consists of a histidine at position 153, an aspartic acid at position 206, and a threonine, the most critical protease catalytic site of TSP50, at position 310. In the peptidase S1 family, there is usually a serine. In this way, TSP50 could represent a new kind of serine protease. The catalytic triads of TSP50 were found to be located near the opening of the pocket, which is formed by two groups of β sheet structures. This structure may provide the proteins with selective access to the threonine catalytic site of TSP50.

### Acquisition of Cell Strains Stably Expressing TSP50 and TSP50 T310A Mutation

TSP50 contains a distinctive catalytic triad and the threonine catalytic site within this catalytic triad is crucial for its protease activity [2], [11]. Besides protease activity, TSP50 has also been shown to facilitate cell proliferation [5], [6]. To determine whether protease activity is essential to TSP50-mediated promotion of cell growth, we established a point mutant construct of TSP50, TSP50 T310A, a protease-dead mutant [11].

**Figure 2:**
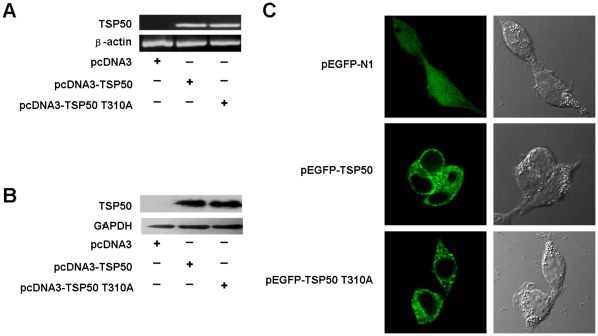
Analysis of TSP50 and TSP50 T310A expression and cellular localization. (A) TSP50 mRNA levels in pcDNA3 cells, pcDNA3-TSP50 cells and pcDNA3-TSP50 T310A cells were analyzed using RT-PCR. β-actin was used as an internal control to check the efficiency of cDNA synthesis and PCR amplification. (B) TSP50 protein levels in pcDNA3 cells, pcDNA3-TSP50 cells and pcDNA3-TSP50 T310A cells were analyzed by Western blotting. Glyceraldehyde-3-phosphate dehydrogenase (GAPDH) was used as a loading control. (C) Cellular localization of TSP50 and TSP50 T310A were determined in 293T cells transiently transfected with either pEGFP-TSP50 or pEGFP-TSP50 T310A by confocal microscopy. The right and left panels show the fluorescent and bright fields respectively. pEGFP-N1 empty vector was used as control.

Our previous study has shown that overexpression of TSP50 in CHO cells markedly promoted cell proliferation and colony formation in vitro. To determine the effects of point mutation on TSP50-mediated promotion of cell growth, plasmids carrying either a wild-type TSP50 gene or the mutant were transfected into CHO cells. Stably transfected cell lines were obtained by G418 selection. The same cell line stably transfected with empty plasmid was used as a negative control, here called pcDNA3 cells. The cell line stably transfected with wild-type TSP50 is here called pcDNA3-TSP50 cells. Stable cell line carrying the TSP50 mutant was named after its mutated form, pcDNA3-TSP50 T310A cells. RT-PCR (Figure 2A) and Western blot analysis (Figure 2B) confirmed the mRNA and protein expression of various TSP50s in the stably transfected cells. The results showed that the levels of all TSP50 proteins were similar. In this way, these cell strains showing stable expression of TSP50 and its point mutant were available for further functional analysis. Prior study has shown that TSP50 was located in the ER and cytoplasmic membrane [11]. To determine whether there had been any change in cellular localization of the mutant TSP50, pEGFP-N1, pEGFP-TSP50, and pEGFP-TSP50 T310A were transfected into 293T cells. They were monitored by confocal microscopy. As shown in Figure 2C, pEGFP-TSP50 and pEGFP-TSP50 T310A showed similar cellular localization and the mutation was not found to change the cellular localization of TSP50.

### Effects of Point Mutation TSP50 T310A on TSP50-mediated Promotion of Cell Growth

**Figure 3:**
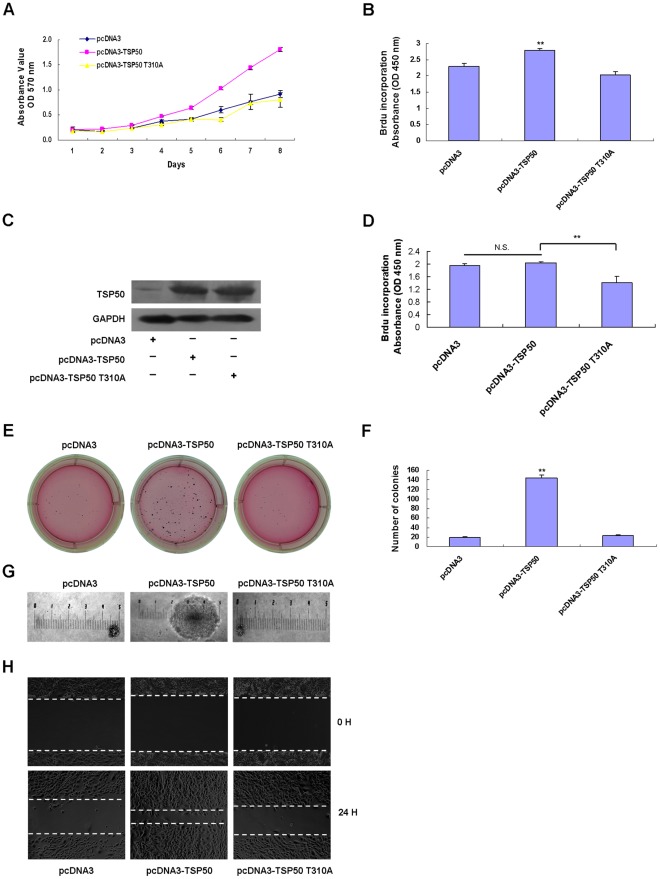
Promotion of cell proliferation by TSP50 is abolished by T310A point mutation. (A) The effects of TSP50 and TSP50 T310A on cell proliferation of CHO cells were determined by MTT assay. The experiments were repeated three times with similar results. (B) Effects of TSP50 and TSP50 T310A on cell proliferation of CHO cells were determined by BrdU incorporation assay. Results are representative of three independent experiments. (C) TSP50 protein levels in pcDNA3 L02 cells, pcDNA3-TSP50 L02 cells, and pcDNA3-TSP50 T310A L02 cells were analyzed by Western blotting. GAPDH was used as a loading control. (D) Effects of TSP50 and TSP50 T310A on cell proliferation of human hepatic L02 cells were determined by BrdU incorporation assay. Results are representative of three independent experiments. (E) Effects of TSP50 and TSP50 T310A on colony formation ability of CHO cells were determined by soft agar colony formation assays. Viable colonies were stained with 5 mg/ml of MTT 15 days after plating, and then the plates were photographed. (F) The number of colonies with diameters over 1 mm in each dish was determined. **indicates *P* < 0.01 with respect to the value for pcDNA3 cells. (G) The representative colony size of each experimental group is shown. (H) Scratch-wound assay on fibronectin-coated plates. Cells were seeded into fibronectin-coated (10 µg/ml) six-well plates in DMEM and cultured overnight. Photographs were taken 24 h after the wound was made. The experiment was repeated three times and similar results were obtained each time.

To compare the effects of wild-type TSP50 to those of its mutant on cell proliferation, we evaluated the cell proliferation capacity of stable cell lines expressing different TSP50s. MTT cell proliferation assay showed that, compared to negative pcDNA3 cells, pcDNA3-TSP50 cells grew very quickly. This was consistent with previous reports. However, the growth rate of pcDNA3-TSP50 T310A was similar to that of pcDNA3 cells (*P*>0.05) (Figure 3A). BrdU incorporation assay also showed that the increased uptake of BrdU by overexpression of TSP50 was eliminated by the point mutant TSP50 T310A (Figure 3B). We observed similar results in other cell lines. Plasmids carrying either a wild-type TSP50 gene or the mutant were also transfected into human hepatic L02 cells and a BrdU incorporation assay was performed. Human hepatic L02 cells express TSP50 endogenously (Figure 3C) and pcDNA3-TSP50 L02 cells grew slightly faster than pcDNA3 L02 cells (Figure 3D). However, TSP50 T310A severely restrained the growth of human hepatic L02 cells (Figure 3D).

Colony formation assays allow simple and direct assessment of the anchorage-independent growth capacity of colony-forming cells [12], [13], [14]. As indicated in Figures 3E, 3F, and 3G, pcDNA3-TSP50 cells formed much larger colonies than those formed by pcDNA3 cells, whereas the colonies formed by pcDNA3-TSP50 T310A cells were similar to those formed by pcDNA3 cells. Taken together, these data indicate that the point mutation TSP50 T310 can abolish the cell-growth-promoting activity of TSP50, suggesting that protease activity is required for TSP50 to promote cell proliferation.

It had been shown that the growth rate of transplanted CHO cells depends on the level of fibronectin receptor expressing on the cell surface [15]. Considering that TSP50 is associated with cellular membranes, this suggests that alsoTSP50 exerts its enzymatic activity on the cell membrane, where it can cleave ECM-receptors and other targets [11]. To address the functional connection between expression of the fibronectin receptor and activity of TSP50, we performed scratch-wound assay on fibronectin-coated plates. As shown in Figure 3H, migration of pcDNA3-TSP50 cells into the gap was much quicker than that of pcDNA3 cells. However, wound closure was greatly inhibited in the pcDNA3-TSP50 T310A cells (Figure 3H). Considering that the mutation did not change the cellular localization of TSP50 (Figure 2C), these data indicate that TSP50, as a threonine protease, may also exert its cell-growth-promoting function partially through cleavage of ECM receptors.

### Analysis of the Pro-proliferative and Anti-apoptotic Function of TSP50 Based on the Flow Cytometry Analysis

**Figure 4:**
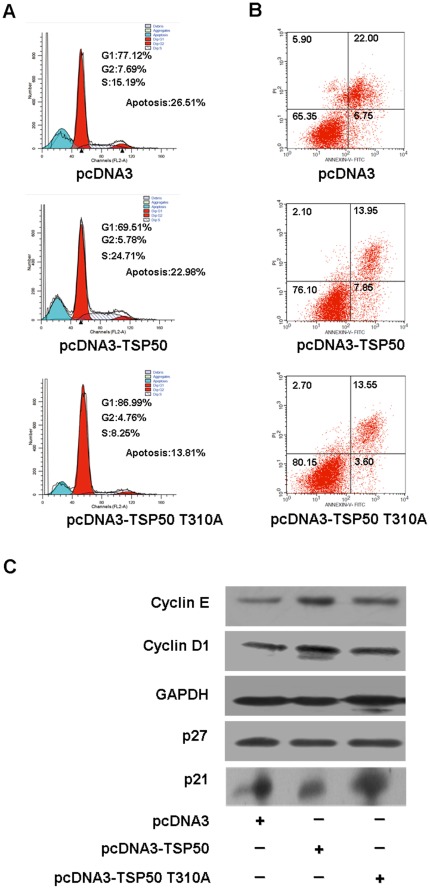
Analysis of the pro-proliferative and anti-apoptotic function of TSP50 based on the flow cytometry analysis. (A) Cell cycle analysis. Flow cytometric detection of cell cycle by propidium iodide fluorescence were conducted in pcDNA3 cells, pcDNA3-TSP50 cells and pcDNA3-TSP50 T310A cells harvested after 96 h of growth in complete medium. (B) Annexin V/PI staining assay. pcDNA3 cells, pcDNA3-TSP50 cells, and pcDNA3-TSP50 T310A cells were grown for 96 h in complete medium, stained with Annexin V and propidium iodide, and analyzed by flow cytometry. (C) Effects of TSP50 and TSP50 T310A on the levels of cell cycle regulatory proteins. The cyclin E, cyclin D1, p27, and p21 levels in pcDNA3 cells, pcDNA3-TSP50 cells, and pcDNA3-TSP50 T310A cells were detected by Western blotting. GAPDH was used as a loading control.

Upon reaching confluency, CHO cells showed no contact-induced growth arrest, but they did undergo high-density dependent apoptosis [16]. Figure 3A shows that, until day 4 there was no significant difference in the growth kinetics of stable transfected CHO cells. However, the difference became obvious after day 4, indicating that TSP50 might protect cells from high-density induced apoptosis. To strengthen the proposed pro-proliferative function of TSP50 and to confirm (or exclude) its anti-apoptotic effects, we undertook a careful analysis of cell cycle progression and apoptosis in the cells grown for 96 h in complete medium. Flow cytometry analysis showed that wild-type TSP50 could accelerate the G1-S transition and protect CHO cells from cell-density-dependent apoptosis (Figure 4A and 4B). However, TSP50 T310A was found to abolish the pro-proliferative function of TSP50, as indicated by the lack of acceleration of the G1-S transition in CHO cells transfected with TSP50 T310A, but it can still protect CHO cells from cell-density-dependent apoptosis (Figure 4A and 4B). This implies that protease activity of TSP50 is essential for its pro-proliferative function but not for enhancing its resistance to density-induced apoptosis.

We also detected levels of some cell cycle regulatory factors in cells expressing TSP50 and cells expressing TSP50 T301A. Consistent with the flow cytometry analysis of the cell cycle shown in Figure 4A, overexpression of TSP50 was found to elevate the expression of cyclin D1 and cyclin E, which can accelerate the G1-S transition. In addition, the expression of p27 and p21, which can inhibit the activity of all G1 cyclin/cdk complexes, was suppressed in pcDNA3-TSP50 cells. However, the TSP50 T310A mutation abolished all of these effects (Figure 4C).

### Effects of Point Mutation TSP50 T310A on TSP50-mediated Resistance to DOX-induced Apoptosis

**Figure 5:**
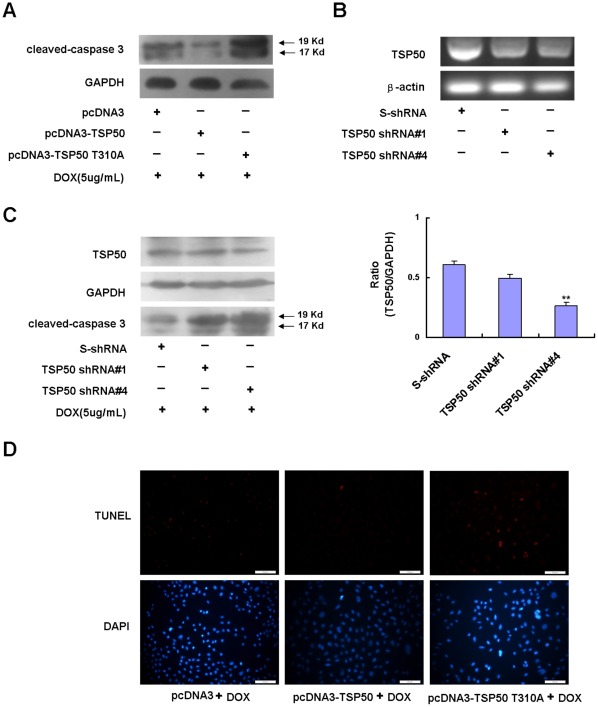
Effects of T310A point mutation on TSP50-mediated resistance to DOX-induced apoptosis. (A) pcDNA3 cells, pcDNA3-TSP50 cells, and pcDNA3-TSP50 T310A cells were treated with 5 µg/ml of DOX for 12 h, and cleaved-caspase 3 protein levels in these cells were detected by Western blotting. GAPDH was used as a loading control. (B) S-shRNA, TSP50 shRNA#1, and TSP50 shRNA#4 were transfected into pcDNA3-TSP50 cells, and the TSP50 mRNA levels of these cells were analyzed by RT-PCR. β-actin was used as an internal control to check the efficiency of cDNA synthesis and PCR amplification. (C) S-shRNA, TSP50 shRNA#1, and TSP50 shRNA#4 were transfected into pcDNA3-TSP50 cells, and cleaved-caspase 3 and TSP50 protein levels in these cells were detected by Western blotting after pretreatment with 5 µg/ml of DOX for 12 h. GAPDH was used as a loading control. Quantification of WB for TSP50 levels using ImageJ software is shown on the left panel. (D) Cells were treated with 5 µg/ml of DOX for 12 hours and stained using the TUNEL Assay Kit (Cat. no 12 156 792 910 Roche Applied Sciences, Mannheim, Germany).

Doxorubicin (DOX), a widely used chemotherapeutic agent, can induce apoptosis in various types of cancer cells [17], [18]. Previous studies have shown that knockdown of TSP50 by RNA interference can result in increased sensitivity to DOX-induced apoptosis in P19 cells [5]. We then investigated whether overexpression of TSP50 could enhance the resistance to DOX-induced apoptosis in CHO cells and whether this activity could be abolished by point mutation TSP50 T310A. As shown in Figure 5A, overexpression of TSP50 inhibited DOX induced activation of caspase-3, while knockdown of TSP50 could reverse the sensitivity of CHO cells to DOX-induced apoptosis (Figures 5B and 5C). TUNEL assay also confirmed that pcDNA3-TSP50 cells could enhance the resistance of CHO cells to DOX-induced apoptosis and that this effect could be abolished by the point mutation T310A (Figure 5D).

### Abolishment of TSP50-mediated Tumorigenicity by Point Mutation TSP50 T310A

**Figure 6:**
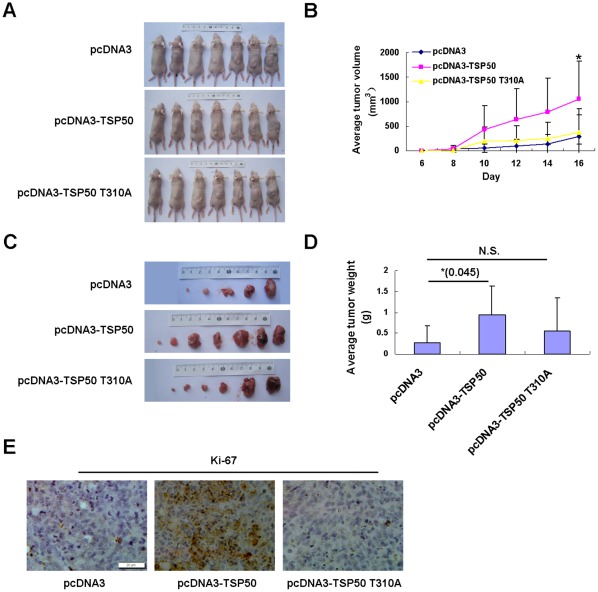
Promotion of tumor growth in NOD/SCID mice by TSP50 is abolished by T310A mutation. 5×10^6^ cells of indicated cells were suspended in physiological saline and injected subcutaneously into the back of the cohorts of 7 BALB/C-scid mice. After 16 days, mice were killed and tumors were removed. (A) The photographs of all the NOD/SCID mice from each group at the end of the experiments. (B) Average tumor volume of each group (n = 7). * Student’s t-test analysis indicated that there was statistical significance in differences between pCDNA3-TSP50 group and pCDNA3 group. (C) Photographs of all the dissected tumors from each group. (D) Average tumor weight of each group. Statistical analysis was performed using the Student’s t test. (E) Immunohistochemical analysis of Ki67 expression in histological sections of tumors from each experimental group. Scale bar: 20 µm.

Because TSP50 T310A mutation can abolish the activity of TSP50 to promote cell growth, we then determined whether they also affect the tumorigenicity of TSP50. We injected 5×10^6^ cells from each stable CHO cell line expressing either wild-type or point mutant TSP50 into the subcutaneous tissue of each athymic (nude) mouse. As in previous studies, the growth rate of tumors derived from pcDNA3-TSP50 cells were faster than that of tumors originating from pcDNA3 cells (Figures 6A and B) and the tumors in pcDNA3-TSP50 cells were heavier as well (Figures 6C and D). However, the growth rates and the weights of tumors derived from pcDNA3-TSP50-T310A-expressing cells were similar to those in pcDNA3 cells (*P*>0.05). Next, tumors from each group were subjected to immunohistochemical analysis with antibodies against Ki67. The results showed that TSP50-dependent hyperproliferation is impaired in tumors derived from CHO cells expressing TSP50 T310A (Figure 6E). These observations provide strong evidence that the ability of TSP50 to promote cell proliferation is abolished by the TSP50 T310A point mutation.

### Abolishment of the Interaction of TSP50 with NF-κB:IκBα Complex and Effects on NF-κB Signaling

Increasing evidence suggests that the NF-κB signaling pathways play important roles in inflammation and tumor development [19], [20], [21], [22]. It has been shown that inflammation and tumorigenesis are closely linked processes [23], [24], [25]. In mammals, the NF-κB family is composed of five related transcription factors, p50, p65 (also RelA), p52, c-Rel, and RelB. These transcription factors share an N-terminal DNA-binding/dimerization domain, called the Rel homology domain, through which they can form homo- and heterodimers. In most cells, NF-κB dimers are inactive, residing predominantly in the cytoplasm in a complex with inhibitory IκB proteins (IκBα, IκBβ, IκBε, IκBζ, p100, p105, Bcl3, and IκBns). When signaling pathways are activated, the IκBs protein is degraded and NF-κB dimers enter the nucleus to modulate target gene expression [26], [27].

**Figure 7:**
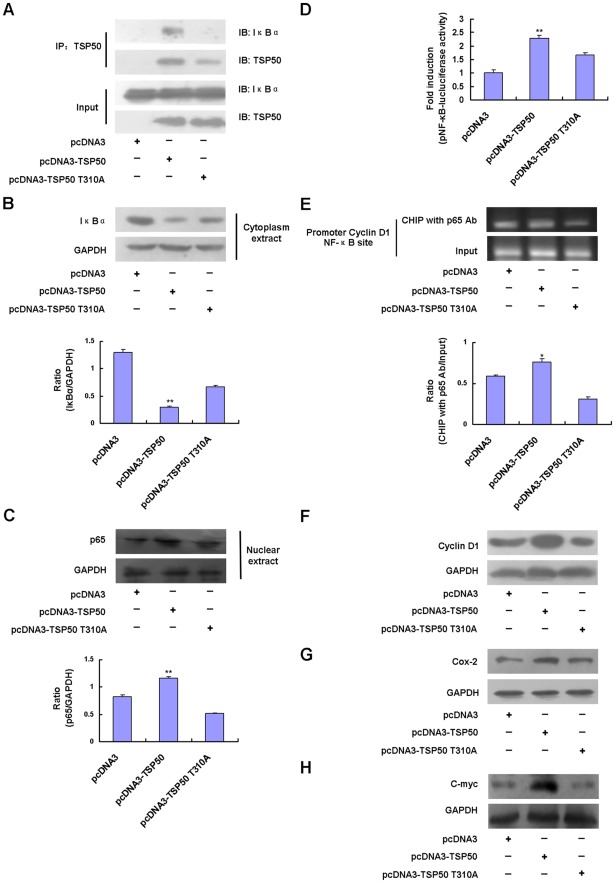
T310A mutation decreases TSP50’s ability to interact with NF-κB:IκBα complex and to promote NF-κB signaling. (A) Interaction between TSP50 and TSP50 T310A with NF-κB:IκBα complex. Equal amounts of protein from the cytoplasmic fraction of cells and immunoprecipitates with anti-TSP50 antibodies were subjected to immunoblotting with antibodies as indicated. (B) and (C) Effects of TSP50 or TSP50 T310A on NF-κB activation. pcDNA3 cells, pcDNA3-TSP50 cells and pcDNA3-TSP50 T310A cells were treated with 100 ng/ml of PMA for 15 min, and then (B) IκBα protein levels in these cells or (C) p65 protein levels in the nuclear fractions were detected by Western blotting, and quantitated using ImageJ software. GAPDH was used as a loading control. Experiments were carried out in triplicate. (D) NF-κB luciferase reporter assay. HEK293T cells were transiently transfected with pNF-κB-luc plasmid along with pcDNA3-TSP50, pcDNA3-TSP50 T310A, or pcDNA3 empty vector, respectively. Forty-eight hours after transfection, cells were pretreated with 400 ng/ml of PMA for 15 min and luciferase activity was assayed. The results were expressed as the fold activity in the luciferase activity of each sample versus that of the vector control. Three independent replicates of each experiment were performed. (E) NF-κB CHIP assay. L02 cells transfected with pcDNA3 empty vector, pcDNA3-TSP50, and pcDNA3-TSP50 T310A were treated with 400 ng/ml of PMA for 15 min before harvesting. The anti-p65 antibodies were used for immunoprecipitation and cyclin D1 promoter fragments were amplified and quantitated using ImageJ software. (F) Effects of TSP50 and TSP50 T310A on the PMA-induced expression of NF-κB target gene. L02 cells transfected with pcDNA3 empty vector, pcDNA3-TSP50 or pcDNA3-TSP50 T310A were treated with 100 ng/ml of PMA for 4 h, and then cytoplasmic extracts were analyzed by Western blotting using antibodies against cyclinD1 and GAPDH. (G) and (H) Effects of TSP50 or TSP50 T310A on the PMA-induced expression of NF-κB target genes. pcDNA3 cells, pcDNA3-TSP50 cells, and pcDNA3-TSP50 T310A cells were treated with PMA (100 ng/ml) for 4 h, and then cytoplasmic extracts were analyzed by Western blotting using antibodies against (G) COX-2, (H) C-myc, and GAPDH.

Our prior studies have shown that TSP50 can interact with the NF-κB:IκBα complex and activate the NF-κB signaling pathway. This could be one of the mechanisms underlying TSP50-mediated promotion of cell proliferation [6]. To determine how the TSP50 T310A mutation abolishes the effects of TSP50 on cell proliferation, the ability of the mutant TSP50s to interact with NF-κB:IκBα complex and to promote NF-κB signaling were examined. TSP50 T310A mutants were not found to interact with the NF-κB:IκBα complex under the same conditions as wild-type molecules (Figure 7A). This strongly suggests that TSP50 needs to combine with the NF-κB:IκBα complex to promote cell proliferation. Immunoblotting showed that the degradation of IκBα in pcDNA3-TSP50 T310A cells was delayed (Figure 7B), and the translocation of NF-κB p65 to the nucleus was decelerated after PMA treatment compared with pcDNA3-TSP50 cells (Figures 7C). PMA is an established NF-κB activator [28]. Furthermore, NF-κB signaling activity reporter assay also showed that the T310A mutation dramatically abolished the enhancement of pNF-κB-luc luciferase activity by TSP50 (Figure 7D). We also performed chromatin immunoprecipitation (ChIP) to analyze the recruitment of p65 to the cyclin D1 promoter, which has NF-κB binding sites. Results showed that the recruitment of p65 to the cyclin D1 promoter was more pronounced in L02 cells transfected with pcDNA3-TSP50 than in L02 cells transfected with pcDNA3 empty vector. However, TSP50 T310A blocked the recruitment of p65 to the cyclin D1 promoter (Figure 7E). Next, we investigated the protein levels of some NF-κB target genes in both TSP50- and TSP50 T301A- expressing cells. As shown in Figures 7F, 7G, and 7H, overexpression of TSP50 enhanced the expression of cyclin D1, Cox-2, and C-myc after cells had been pretreated with PMA for 4 h. However, overexpression of TSP50 T310A failed to enhance PMA-induced expression of these NF-κB target genes (Figures 7F, 7G, and 7H). These observations provide strong evidence that TSP50 needs to combine with NF-κB:IκBα complex to promote cell proliferation and T310A mutation impairs TSP50’s ability to facilitate cell proliferation at least partly through destroying TSP50’s abilities to interact with NF-κB:IκBα complex.

### Reductions in the Flexibility of the Pocket-edge Weaken TSP50’s Ability to Interact with NF-κB:IκBα Complex

**Figure 8:**
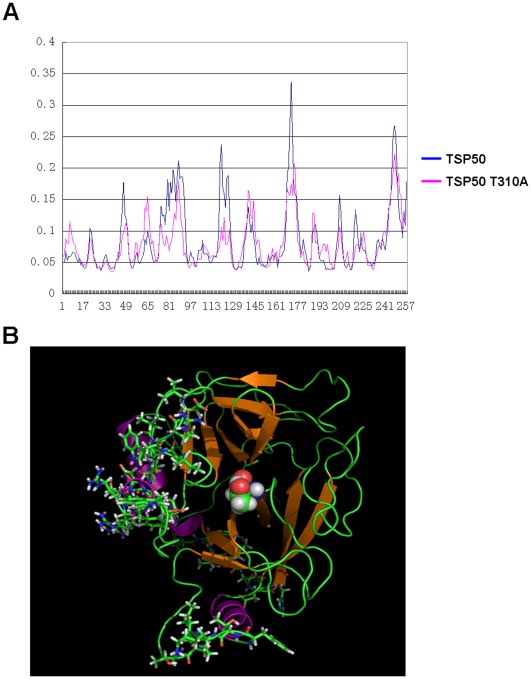
Reductions in the flexibility of the pocket-edge weaken TSP50’s ability to interact with NF-κB:IκBα complex. (A) The RMSF values of every αC atom of TSP50 and TSP50 T310A models were calculated. (B) Distribution of sharp reductions of flexibility residues, represented as sticks, is shown, and spheres represent Thr 310. This diagram was prepared using PyMOL.

The co-immunoprecipitation assay showed that mutated TSP50 T310A could not interact with the NF-κB:IκBα complex (Figure 7A). To determine how mutation of threonine 310 structurally blocks TSP50-IkBa:NF-kB interactions, we performed a molecular dynamics simulation focusing on the structural modifications caused by the mutation. We noted that the mutated model presented some changes in flexibility along the edge of the active pocket, so we calculated the RMSF value of every αC atom in both models. We found several sharp reductions in residue flexibility in the mutated model, and these reductions were enriched in residues No. 44–48, 74–86, 118–125, and 168–173 (Figure 8A). Advanced analysis indicated that notable parts of these residues were located near the edge of the active pocket of the TSP50 model (Figure 8B).

## Discussion

Serine proteases have demonstrated roles in tumor growth [29], [30]. For example, there is a strong correlation between urokinase plasminogen activator levels and greater metastatic potential in breast cancer [31]. TSP50, a newly discovered threonine protease, contains amino acid sequences and enzymatic structures similar to those of many serine proteases. It has been shown that overexpression of TSP50 can promote cell proliferation and colony formation in vitro and stimulate tumor formation in nude mice [6]. It has also been shown that knockdown of TSP50 in mouse P19 cells can inhibit tumor cell proliferation and induce apoptosis [5]. Further understanding of TSP50 biology may provide additional mechanistic insights into role of serine proteases in cell proliferation.

As a novel threonine protease, the catalytic triad of TSP50, especially Thr310, is crucial for its protease activity [11]. In this study, we show that the TSP50 T310A mutation can impair the ability of TSP50 to promote cell proliferation, colony formation, and tumorigenicity (Figures 3 and 6). This provides strong evidence that the threonine protease activity of TSP50 is pivotal to TSP50-dependent hyperproliferation, anchorage-independent growth, and tumor formation.

Previous research has shown that knockdown of TSP50 by RNA interference can strengthen DOX-induced apoptosis in P19 cells [5]. Here we showed that overexpression of TSP50 can enhance resistance to DOX-induced apoptosis in CHO cells while overexpression of TSP50 T310A cannot (Figures 5A and 5D). This indicates that the threonine protease activity of TSP50 is also important for the anti-apoptotic function of TSP50. In addition, flow cytometry analysis (Figures 4A) showed that TSP50 overexpression reduced high-density induced apoptosis from 26.51% to 22.98%. This implied that overexpression of TSP50 could also enhance resistance to high-density-induced apoptosis. However, although overexpression of TSP50 T310A could not enhance the resistance to DOX-induced apoptosis in CHO cells (Figures 5A and 5D), overexpression of TSP50 T310A seem to protect CHO cells from high-density induced apoptosis (13.81%) relative to pcDNA3 cells (26.51%). This may be because pcDNA3-TSP50 T310A cells grew too slowly to induce high-density dependent apoptosis. The S stage of pcDNA3 cells is 15.19%, but that of pcDNA3-TSP50 T310A cells is only 8.25% (Figure 4A).

Our recent studies have shown that TSP50 can interact with NF-κB:IκBα complex and promote NF-κB signaling pathway to facilitate cell proliferation [6]. Here, we found that mutant TSP50 T310A lost its ability to interact with NF-κB:IκBα complex under certain conditions. Furthermore, mutant TSP50 T310A affects IκBα degradation and NF-κB p65 translocation into the nucleus compared with TSP50 expressing cells (Figure 7). These results indicate that IκBα could be a major target of TSP50 and that TSP50 may increase IκBα degradation by direct interaction. In addition, the TSP50 T310A mutation exerts a dominant-negative effect, at least partially through destroying the ability of TSP50 to interact with the NF-κB:IκBα complex and further affects the enhancement of IκBα degradation and NF-κB p65 nuclear translocation facilitated by TSP50.

We have also shown several sharp reductions in residue-flexibility in the mutated model. Notable parts of these residues are located near the edge of the active pocket of the TSP50 model (Figure 8). Considering that these pocket-edge residues have large side chains, we suspected that the reduction of the flexibility of the pocket-edge might weaken the capability of the active pocket edge to fit the substrates.

In conclusion, the results obtained here indicate that the threonine protease activity of TSP50 is essential to its function in hyperproliferation, anchorage-independent growth, tumor formation, and DOX-induced apoptosis. In addition, the T310A mutation exerts its dominant-negative effect at least partially through the destruction of TSP50’s ability to interact with NF-κB:IκBα complex. This affects IκBα degradation and NF-κB p65 translocation into the nucleus compared with TSP50 expressing cells. These findings lend insight into the mechanism by which TSP50 enhances cell proliferation though its protease activity. The dominant negative mutant allele constructed in this report may further our understanding of the TSP50 gene.

## Materials and Methods

### Ethical Treatment of Animals

This study was carried out in strict accordance with the recommendations in the Guide for the Care and Use of Laboratory Animals of the National Institutes of Health. The protocol was approved by the Committee on the Ethics of Animal Experiments of Northeast Normal University (Permit Number: SCXK 2009-0004). All surgery was performed under sodium pentobarbital anesthesia, and all efforts were made to minimize suffering.

### Cell Lines and Cell Culture

Chinese hamster ovary (CHO) cells, human embryonic kidney (HEK293T) cells and human hepatic L02 cells were obtained from Cell Bank of the Chinese Academy of Sciences (Shanghai, China). The cells were maintained in Dulbecco’s modified Eagle’s medium (DMEM, Gibco), supplemented with 10% fetal bovine serum (FBS, TBD, China) and antibiotics (100 U/ml penicillin and 100 µg/ml streptomycin).

### Plasmids

The TSP50 and its point mutation construct were cloned into the pcDNA3 basic vector and pEGFP-N1 vector. The point mutation construct pcDNA3-TSP50 T310A, originating from the construct pcDNA3-TSP50, was amplified by PCR. The point mutation construct was created using a Site Directed Mutagenesis Kit (Beyotime, Jiangsu, China). The favorable mutagenesis efficiency and low error rate of the method render the analysis of large numbers of subclones unnecessary [32]. The primer sequences used were as follows: TSP50 T310A-For GTTCTGCTATGAGCTAGCTGGAGAGCCCTTGGTC, Rev GACCAAGGGCTCTCCAGCTAGCTCATAGCAGAAC. To create the TSP50 shRNA expression vector, the pRNAT-U6.1/Hygro vector (GenScript Corporation, NJ, U.S.) was used for DNA vector-based shRNA synthesis. pRNAT-U6.1/Hygro expresses hairpin sequences which utilize the U6 RNA pol III promoter, and it also has a coral GFP marker (cGFP) under CMV promoter control. For each target, the sense and antisense strands were separated by a loop that comprised nine nucleotides (5'-TTCAAGAGA-3') and a polythymidine tract to terminate transcription. The shRNA sequences targeting TSP50 were TSP50 shRNA#1 AAGTTCTGCTATGAGCTAACT and TSP50 shRNA#4 GAGTGTGACAATTTCTACC. The sequence of the scrambled shRNA negative control (GenScript Corporation, NJ, U.S.) was GACGCTTACCGATTCAGAA. This sequence has no significant homology to any known mouse or human gene sequence.

### Antibodies

Rabbit polyclonal antibodies against IκBα, p27, cyclin E, COX-2, Ki67 and mouse monoclonal antibodies against p65, p21, cyclin D1, and C-myc were obtained from Santa Cruz Biotechnology (Santa Cruz, CA, U.S.). Rabbit polyclonal antibody against cleaved caspase-3 was obtained from Cell Signaling Technology Inc. (Beverly, MA, U.S.), and mouse monoclonal antibody against GAPDH was purchased from Kangcheng Biotech (Shanghai, China). Anti-TSP50 monoclonal antibody was prepared in our laboratory [33].

### Modeled Structure of TSP50

The one-letter amino acid sequence of TSP50 was obtained from a protein sequence database hosted by NCBI. Automatic homology modeling was attempted as a routine approach to the 3D structure of TSP50. NCBI-blast was performed, and we observed a high degree of homology between the serine protease family and TSP50.

Protein-model-building protocols, such as I-TASSER, THREADER, HH-pred, and MODEL were used to produce 3D-structure of TSP50, with the hope of gaining a reliable result [34], [35], [36]. Models with less probability were filtered out according to the performance of fitting to some generally accepted information about the characteristics of the active-site-based-region of the serine protease family. At the end of the experiment, the model built using the HH-pred web server to predict sequence-alignments and MODEL to translate the alignments into 3D-structure was found to be the best.

### Stable Transfection

Transfections were performed using LipofectamineTM2000 (Invitrogen, CA, U.S.) according to the manufacturer’s instructions. 5×10^5^ CHO cells were seeded in each well of a 6-well plate 24 hours before transfection. The cells were transfected with 5 mg of a wild-type TSP50 gene construct or the mutation construct. The same cell line transfected with pcDNA3 empty plasmid was used as a negative control. Transfected cells were incubated in the presence of G418 for 2 weeks and stable transfected cell lines were obtained.

### RNA Extract and RT-PCR

Total RNA was isolated from cultured cells using Trizol reagent (Invitrogen, Carlsbad, CA, U.S.) according to the manufacturer’s instructions. RNA was quantified by measuring the absorbance (A260 nm) and stored at –80°C until use. One microgram of total RNA was reverse transcribed with oligo (dT) primers using a reverse transcription system (TAKARA, Dalian, China). The single-stranded cDNA was amplified by PCR using TSP50-specific primer and β-actin primer pairs. PCR was performed for 30 cycles (each cycle consisting of 94°C for 30 s, 54°C for 30 s, and 72°C for 30 s). The PCR products were analyzed by electrophoresis on a 1% agarose gel. Primer sequences used were as follows: TSP50-For CGGATCCATGCAGGGGAAGCC, Rev GCTCTAGAAGTCAGAGGGCAG; β-actin-For TCGTGCGTGACATTAAGGAG, Rev ATGCCAGGGTACATGGTGGT.

### Western Blot Analysis

Cells were harvested and rinsed twice with PBS. Cell extracts were prepared with lysis buffer (1% Nonidet P-40, 50 mM Tris-HCl (pH 7.5), 150 mM NaCl, 1 mM NaF, 1 mM phenylmethylsulfonyl fluoride, 4 µg/ml leupeptin, and 1 µg/ml aprotinin) for 30 min with occasional rocking followed by centrifugation at 12,000 rpm, for 10 min at 4°C. Identical amounts (100 µg of protein) of cell lysate were resolved by 10% sodium dodecyl sulfate-polyacrylamide gel electrophoresis (SDS–PAGE). The resolved proteins were electrophoretically transferred to polyvinylidenefluoride (PVDF) membrane, and blocked with 5% fat-free dry milk in TBST (20 mM Tris-HCl (pH 7.6), 150 mM NaCl, and 0.02% Tween20) for 1 h, at room temperature. The membrane was immunoblotted with indicated antibodies in 1% milk/TBST. To assure equivalent protein loading, the membranes were simultaneously incubated with GAPDH monoclonal antibody (1∶1000) overnight at 4°C. Membranes were washed three times, incubated with HRP-conjugated secondary antibodies for 1 h at room temperature, and washed extensively before detection. The membranes were subsequently developed using ECL reagent (Beyotime, China) and exposed to film according to the manufacturer’s protocol.

### Cellular Localization

pEGFP-TSP50, pEGFP-TSP50 T310A, and the empty vector pEGFP-N1 were transiently transfected into HEK293T cells. After 48 h, the cells were observed and photographed under a confocal microscope.

### Methyl Thiazolyl Tetrazolium (MTT) Assay

Cell proliferation was determined using the MTT assay. Cells were plated (1200 cells/well) in 200 µL of growth medium in 96-well plates in three replicates, and a total of 8 plates were plated. Every 24 h, MTT (20 mM, Sigma Chemical Co.) was added to each well for 4 h. The blue MTT formazan precipitate was dissolved in 100 µL of dimethylsulfoxide (DMSO). The absorbance at 570 nm was measured on a micro-ELISA reader (Bio-Rad, CA, U.S.).

### BrdU Incorporation Assay

The 5-bromo-20-deoxyuridine (BrdU) labeling and detection ELISA kit was purchased from Roche Diagnostics (Mannheim, Germany). DNA synthesis was assessed by measuring incorporation of BrdU into newly synthesized molecules. pcDNA3, pcDNA3-TSP50, and pcDNA3-TSP50 T310A cells were replated at 1×10^3^ cells per well on 96-well plates. Twenty-four hours after plating, BrdU labeling was initiated by adding labeling solution at a final concentration of 10 µM to the culture medium. After 8 h incubation, labeling was stopped. BrdU uptake was measured according to the manufacturer’s instructions.

### Colony Formation Assay

Cells were mixed in 0.35% Noble agar(in DMEM containing 10% fetal bovine serum) and plated at 5000 cells/well onto 6-well plates containing a solidified bottom layer (0.6% Noble agar in the same growth medium). After 15 days, colonies were either unstained or stained with 5 mg/ml MTT (Sigma), and photographed, and three independent soft agar plating experiments were performed for statistical analysis [37].

### Scratch-wound Assay

pcDNA3 cells, pcDNA3-TSP50 cells, and pcDNA3-TSP50 T310A cells were seeded into fibronectin (Cat. no 1030-FN-01M R&D Systems, Inc., 10 µg/ml)-coated six-well plates in 2 ml of DMEM and cultured overnight. Pipette tips were used to scratches in the cell monolayers. Then the monolayers were rinsed and cultured for another 24 h. Pictures were taken at 0 and 24 h using a digital camera system coupled to a microscope.

### Cell Cycle Analysis

Cell cycle analysis based on the flow cytometry was performed using Cell Cycle and Apoptosis Analysis Kit (Cat. no C1052 Beyotime Institute of Biotechnology, China) following the manufacturer’s instructions.

### Apoptosis Assay

Cells were treated with 5 µg/ml of DOX for 12 hours (for TUNEL assay) or grew in complete medium for 96 hours (for detection of Annexin V) and were stained with the TUNEL Assay Kit (Cat. no 12 156 792 910 Roche Applied Sciences, Mannheim, Germany) or Annexin V-FITC Apoptosis Detection Kit (Cat. no KGA106 KeyGEN BioTECH, China) following the manufacturer’s instructions. Cells were then photographed under microscope or analyzed by flow cytometry.

### Tumorigenicity Assay

5×10^6^ cells were suspended in physiological saline and injected subcutaneously into the backs of 7 BALB/C-scid mice. Tumor volumes were determined using calipers. The greatest longitudinal diameter (length) and the greatest transverse diameter (width) were determined. Tumor volume was calculated using the modified ellipsoidal formula [38].

Tumor volume = 1/2(length×width^2^) [39].

### Immunohistochemical Assay

The tumor sections were fixed in 10% buffered formalin, embedded in paraffin, and then deparaffinized and rehydrated using standard procedures. For Ki67 staining, antigen retrieval was performed in citrate buffer (pH 6.0, 30 min) and revealed using a Ki67 rabbit polyclonal antibody. An immunohistochemistry kit (Maixin Bio, China) and DAB (diaminobenzidine) were used as chromagen for Ki67. Negative (omission of the primary antibody and substitution with preimmune serum) controls were included in each slide run.

### Co-immunoprecipitation

The Pierce Crosslink Immunoprecipitation Kit was purchased from Thermo Scientific (U.S.) and the co-immunoprecipitation experiments were performed according to the manufacturer’s instructions.

### Luciferase Reporter Assay

Firefly luciferase activity was measured 48 h after transfection. Analysis of luciferase activity was performed as described previously [11].

### CHIP Assay

CHIP analysis of the cyclin D1 promoter using p65 antibodies in L02 cells was performed 48 h after transfection as described previously [40]. Cells were treated with 100 ng/ml of PMA for 15 min before the analysis. Sequences of primers for amplifying cyclin D1 promoter were as follows: cyclinD1 sense TCCCATTCTCTGCCGGGCTTTGATC; cyclinD1 antisense GCTGGTGTTCCATGGCTGGGGC.

### Statistical Analysis

Data are expressed as means ± SD. Statistical analysis of the data was performed using the Student’s t test. Values of *P* < 0.05 were considered statistically significant.
